# Zebrafish drug screening identifies candidate therapies for neuroprotection after spontaneous intracerebral haemorrhage

**DOI:** 10.1242/dmm.049227

**Published:** 2022-03-29

**Authors:** Siobhan Crilly, Adrian Parry-Jones, Xia Wang, Julian N. Selley, James Cook, Victor S. Tapia, Craig S. Anderson, Stuart M. Allan, Paul R. Kasher

**Affiliations:** 1 Division of Neuroscience and Experimental Psychology, School of Biological Sciences, Faculty of Biology, Medicine and Health, Manchester Academic Health Science Centre, The University of Manchester, Oxford Road, Manchester M13 9PT, UK; 2 Geoffrey Jefferson Brain Research Centre, The Manchester Academic Health Science Centre, Northern Care Alliance and The University of Manchester, Manchester M6 8HD, UK; 3 Division of Cardiovascular Sciences, School of Medical Sciences, Faculty of Biology, Medicine and Health, Manchester Academic Health Science Centre, The University of Manchester, Oxford Road, Manchester M13 9PT, UK; 4 Manchester Centre for Clinical Neurosciences, Salford Royal, NHS Foundation Trust, Manchester Academic Health Science Centre, Stott Lane, Salford M6 8HD, UK; 5 The George Institute for Global Health, Faculty of Medicine, University of New South Wales, Sydney, NSW 2052, Australia; 6 The Biological Mass Spectrometry Core Research Facility, Faculty of Biology, Medicine and Health, The University of Manchester, Manchester M13 9PL, UK

**Keywords:** Zebrafish, Stroke, Intracerebral haemorrhage, ACE inhibitors, Drug screen

## Abstract

Despite the global health burden, treatment of spontaneous intracerebral haemorrhage (ICH) is largely supportive, and translation of specific medical therapies has not been successful. Zebrafish larvae offer a unique platform for drug screening to rapidly identify neuroprotective compounds following ICH. We applied the Spectrum Collection library compounds to zebrafish larvae acutely after ICH to screen for decreased brain cell death and identified 150 successful drugs. Candidates were then evaluated for possible indications with other cardiovascular diseases. Six compounds were identified, including two angiotensin-converting enzyme inhibitors (ACE-Is). Ramipril and quinapril were further assessed to confirm a significant 55% reduction in brain cell death. Proteomic analysis revealed potential mechanisms of neuroprotection. Using the INTERACT2 clinical trial dataset, we demonstrated a significant reduction in the adjusted odds of an unfavourable shift in the modified Rankin scale at 90 days for patients receiving an ACE-I after ICH (versus no ACE-I; odds ratio, 0.80; 95% confidence interval, 0.68-0.95; *P*=0.009). The zebrafish larval model of spontaneous ICH can be used as a reliable drug screening platform and has identified therapeutics that may offer neuroprotection.

This article has an associated First Person interview with the first author of the paper.

## Abstract (precis)

**Summary:** We used a zebrafish larval model to perform drug screening to identify novel and translational compounds for ameliorating brain damage after a haemorrhage, which may support neuroprotective therapeutic development.

## INTRODUCTION

Spontaneous intracerebral haemorrhage (ICH) is a catastrophic neurovascular event that contributes to a considerable global health burden as a leading cause of adult disability (An et al., 2017). Treatment options for ICH are limited to early reversal of anti-coagulants, lowering of blood pressure and, where possible, surgical evacuation of the haematoma. This type of protocol-driven ‘care bundle’ has been shown to reduce 30 day mortality in these patients (Parry-Jones et al., 2019) but may not translate to settings in which access to specialised stroke staff and highly trained surgical teams are limited. Emergency neurosurgery to evacuate the haematoma may only benefit a minority of carefully selected patients, and further work is ongoing to investigate the utility of minimally invasive techniques (Gregson et al., 2012). As such, there is an urgent requirement for a widely accessible and effective medical therapy that can be administered immediately after ICH to ameliorate the brain damage caused by the haematoma and prevent disability that reduces quality of life.

Although mammalian models of ICH have improved our understanding of disease mechanisms, their use as a pre-clinical trialling system for candidate drugs has failed thus far in terms of clinical translation. As such, alternative pre-clinical approaches are required to identify and trial drug compounds (Selim et al., 2018; Withers et al., 2020). Previously, we have shown in a zebrafish larval model that spontaneous ICH from 48 h post-fertilisation (hpf) results in a consistent and characteristic increase in expression of the cell death marker *annexinV* (also known as *annexin a5a/b*) in brain cells that is absent in non-haemorrhaged sibling controls (Crilly et al., 2018; Crilly et al., 2019). Furthermore, these fish exhibit locomotor deficits and neuroinflammatory outcomes comparable to the human condition. Owing to their small size, rapid development and high fecundity, zebrafish larvae offer an attractive pre-clinical system for drug screening (Margiotta-Casaluci et al., 2019; Patton et al., 2021). Zebrafish brain haemorrhage models have previously been used for investigation to identify compounds that can inhibit cerebral bleeding (Yang et al., 2017). However, in this study, larvae were treated after ICH and screened for a reduction in brain injury, which offers a more translationally relevant approach to emergency treatment compared to bleed prevention. To our knowledge, this is the first large-scale study of its kind to employ a spontaneous model of ICH as a platform for drug screening to identify potentially repurposable agents that are neuroprotective.

We have identified 150 compounds that reduced brain injury in the zebrafish model, which could represent new candidate treatments for ICH patients. Further analysis of the successful compounds revealed that two angiotensin-converting enzyme inhibitors (ACE-Is) improved ICH outcomes in the zebrafish larvae. Mass spectrometry identified several key pathways through which ACE-Is might afford protection in ICH, including downregulation of mitochondrial proteins, and upregulation of protein processing and extracellular matrix (ECM)/tight junctions in the blood–brain barrier (BBB), highlighting further research routes for drug development. ACE-Is are widely used to control blood pressure after ICH, but evidence of their use as a specific protective agent is limited. The INTERACT2 trial was a phase III randomised controlled trial of intensive blood pressure lowering in acute ICH, which assessed a range of anti-hypertensives including ACE-Is (Anderson et al., 2013). Overall, no significant reduction in the primary outcome of death or severe disability was observed. In this study, driven by our zebrafish analyses, we undertook secondary analysis of the INTERACT2 dataset to determine the effects of ACE-Is only. Through this sub-analysis, we reveal that ACE-I administered only after ICH is associated with significantly improved patient outcomes, thus validating the zebrafish model and offering new translational insight into the role of ACE-Is as a potential therapeutic for haemorrhagic stroke.

## RESULTS

### Using zebrafish larvae for drug screening

**Fig. 1.**
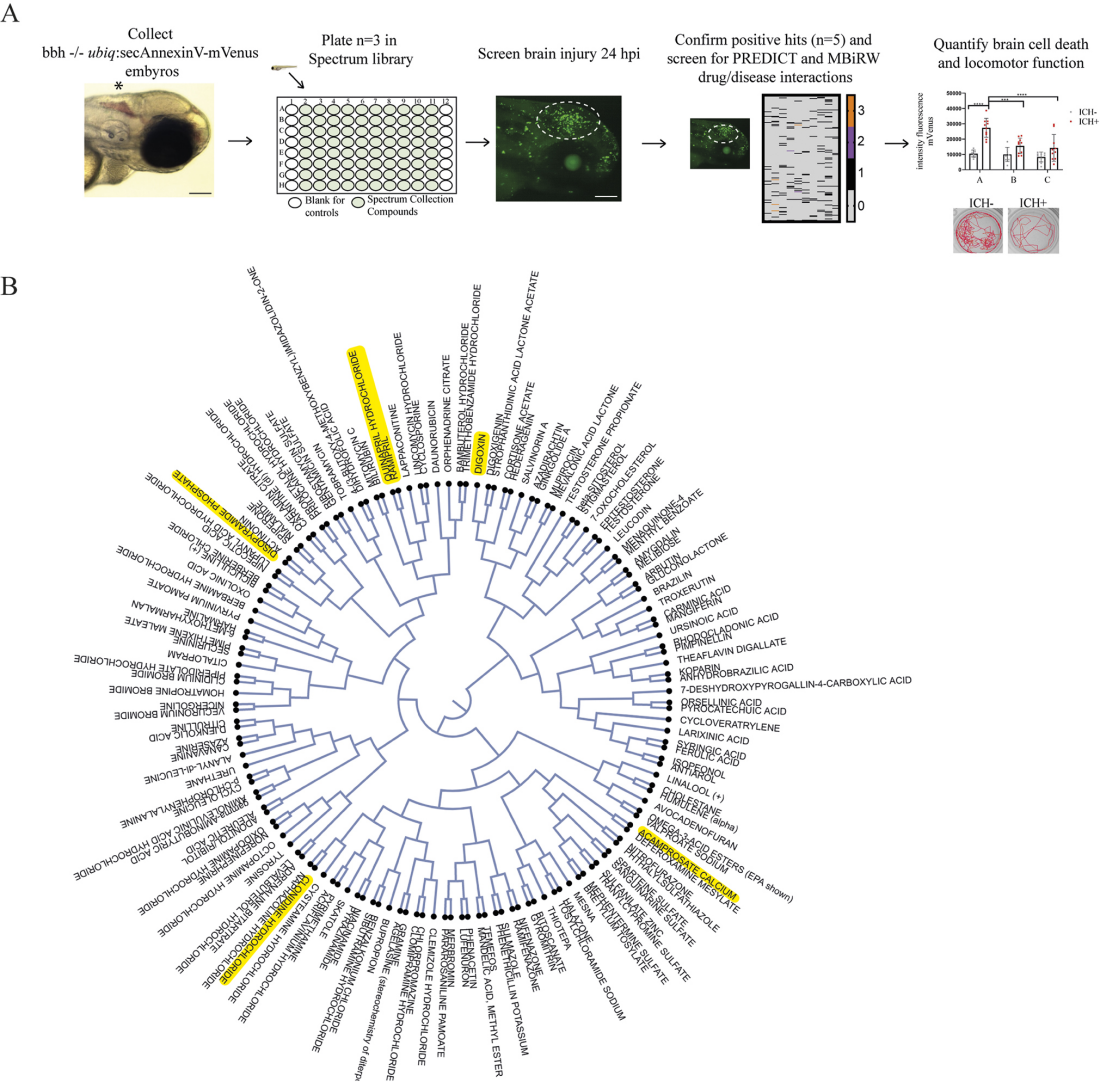
**The drug screen resulted in 150 compounds that prevented brain cell death in a model of ICH.** (A) Schematic of the screening protocol. (B) Structural similarities, based on MACCS keys, between the 150 positive hits represented by a radial tree. The six highlighted compounds were identified as predicted to be efficacious in cardiovascular disease. hpi, h post-injury; ICH, intracerebral haemorrhage; MACCS, Molecular Access System. Scale bars: 100 μm.

To identify novel compounds for ICH therapy, we carried out a manual drug screen using the *bubblehead* (*bbh*; also known as *arhgef7b*) zebrafish larval model of ICH (Crilly et al., 2018; Liu et al., 2007). Transgenic *ubiq*:secAnnexinV-mVenus adults were raised on both a *bbh* mutant and nacre background. Following spontaneous ICH in *bbh* homozygotes at 48 hpf, a significant increase in *annexinV* expression was observed 24 h after injury. Homozygous *bbh* mutants (*n*=3) were screened for clusters of *annexinV*-positive dying cells in the brain at 72 hpf. Compounds capable of reducing brain cell death were repeated (*n*=5) to verify the outcome. Sample sizes were selected based on plate volume. A schematic of the protocol shows the experimental design (Fig. 1A). Full results are available at doi:10.6084/m9.figshare.14096465.

### Screening identified a group of 150 neuroprotective compounds

We identified drugs that had positive outcomes for cerebral oedema, brain cell death and overall larval health. The screen resulted in a 7.5% hit rate (150 compounds) for a reduction in brain cell death 24 h after ICH and no other visible morphological defects. Structural similarities between the 150 compounds were determined using Molecular Access System (MACCS) keys and the cSPADE web application (Ravikumar et al., 2017) (Fig. 1B). In order to refine this number of positive hits for further pre-clinical investigation, we searched both the PREDICT (Gottlieb et al., 2011) and bi-random walk model algorithm (MBiRW) (Luo et al., 2016) datasets for associations between drug and ICH-related cerebrovascular diseases (search terms are listed in Table S1). The PREDICT model is based on the assumption that similarities in drug properties can be used to make associations with diseases with similar phenotypic properties (Gottlieb et al., 2011). Additionally, based on the success of the PREDICT model, the MBiRW calculates novel drug–disease interactions by generating drug–disease similarity networks and combining with known associations (Luo et al., 2016). This resulted in a 0.3% hit rate (six compounds that are highlighted in Fig. 1B: ramipril, quinapril hydrochloride, acamprosate calcium, digoxin, disopyramide phosphate and clonidine hydrochloride). The predicted diseases associated with the six positive drugs are listed in Table 1.

**Table 1.**
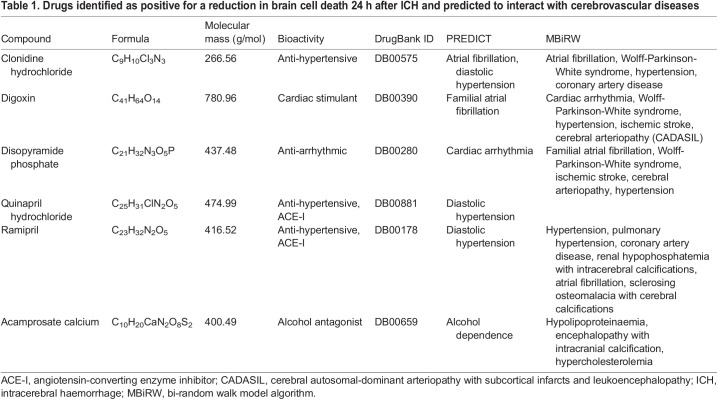
Drugs identified as positive for a reduction in brain cell death 24 h after ICH and predicted to interact with cerebrovascular diseases

### ACE-I treatment offers cellular neuroprotection but does not translate to functionality in the zebrafish

**Fig. 2.**
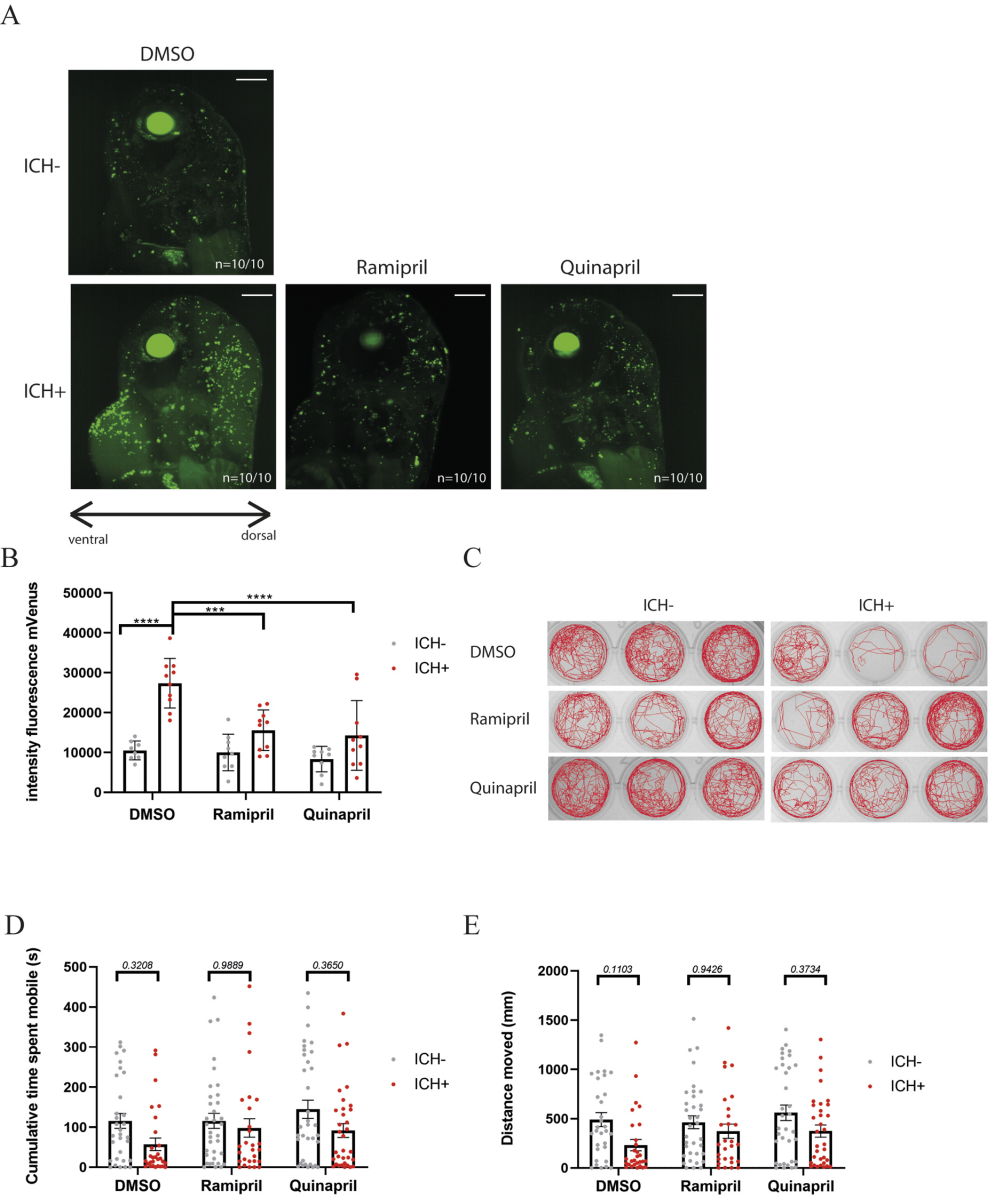
**Angiotensin-converting enzyme inhibitor (ACE-I) treatment offers cellular neuroprotection but does not translate to functionality in zebrafish.** (A) Representative images of *annexinV* expression in the brain at 72 h post-fertilisation (hpf) in DMSO-treated ICH− and ICH+ controls compared to ICH+ individuals that have been treated with ACE-I (*n*=10). (B) *annexinV* expression in the brain 24 h after ICH is significantly increased in DMSO vehicle-treated larvae, as previously observed in this model (***P=0.0003, ****P<0.0001) (Crilly et al., 2018; Crilly et al., 2019). Data (from *n*=10 larvae from three technical repeats) were analysed using a two-way ANOVA and Tukey's post hoc analysis. (C) Representative swimming traces for (*n*=3/24) larvae from each group, tracked over 10 min for spontaneous movement in response to a white-light stimulus every 1 min. Data presented from two technical repeats (clutches). (D,E) Functional analysis of spontaneous locomotion 72 h after ICH (120 hpf) shows an increase in time spent moving (D) and distance moved (E) in ramipril- and quinapril hydrochloride-treated ICH+ groups compared to DMSO vehicle-treated ICH+ groups, although these differences were not significant (*P*-values shown on graph). Data were analysed using mixed linear modelling and chi-squared test for non-parametric data. No significant difference between DMSO ICH+ and treatment ICH+ groups was observed (*P*=0.6); however, the data trend suggests that ICH+ treatment group values are closer to the ICH− treatment group values, highlighted by the increase in *P*-values presented on the graph. Scale bars: 100 μm.

Drug association prediction analysis revealed two ACE-Is within the group of six compounds: ramipril and quinapril hydrochloride. Owing to the wider clinical interest in reducing blood pressure in ICH trials, we decided to continue investigation with these two drugs. Clinical evidence suggests that ACE-Is are protective in ICH cases (Li et al., 2018); however, conclusions are contradictory (Eichel et al., 2010). Verifying that these ACE-Is were effective in the zebrafish model might support potential translatability of other drugs identified by the screen. To confirm a significant reduction in brain cell death in response to ICH, larger samples sizes of larvae (*n*=10 per group) were treated with ramipril or quinapril hydrochloride (25 µM), and brain cell death was quantified as previously described (Crilly et al., 2018). Both drugs showed an average 55% significant reduction in the mVenus intensity fluorescence when compared with dimethyl sulfoxide (DMSO) vehicle-treated ICH+ controls (Fig. 2A,B). To test whether this neuroprotection translated into functional benefits, swimming behaviour was recorded at 120 hpf (Fig. 2C-E). Locomotion was assayed at 120 hpf when there is the most spontaneous swimming, as at earlier time points there are many motionless individuals, which may confound the results. Although not significant at this time point, there is a consistent decrease in swimming distance and total time spent mobile in ICH+ individuals (Crilly et al., 2018). No significant differences in locomotor performance were observed when comparing ICH− and ICH+ individuals in any treatment group (*n*=35); however, a trend towards both increased distance moved and total time spent mobile in the ICH+ groups was observed with treatment.

### Proteomic analysis highlights several pathways associated with neuroprotection after ICH

**Fig. 3.**
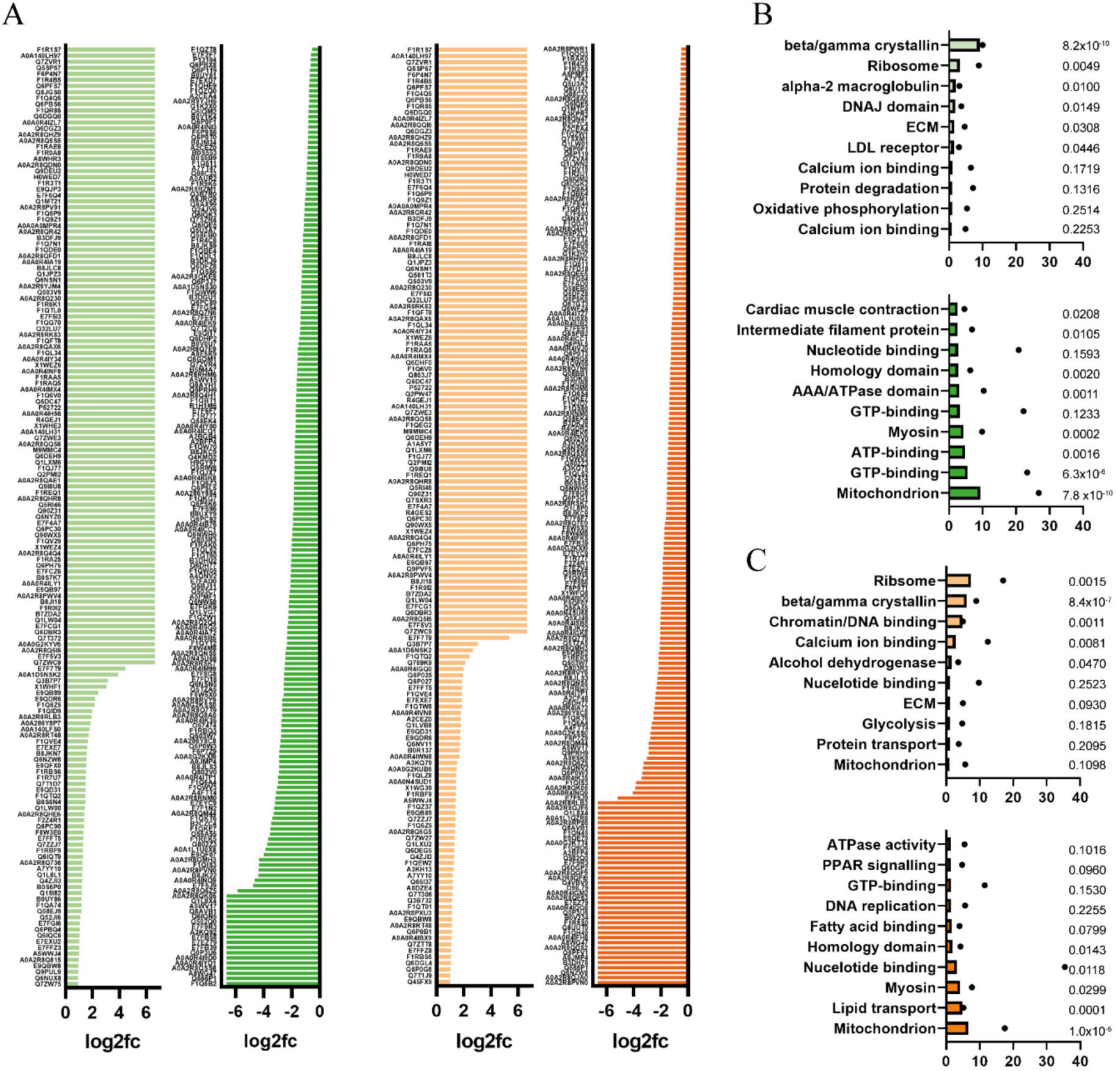
**Proteomic analysis highlights several pathways associated with neuroprotection from ACE-I treatment.** (A) Significant changes in protein expression levels in the heads (*n*=5) of ramipril (left, green)- and quinapril (right, orange)-treated larvae when compared to DMSO controls. (B,C) Functional annotation performed using Database for Annotation, Visualization and Integrated Discovery (DAVID) identified gene ontology, protein pathway and functional category similarities, and the top ten upregulated pathways (top) and downregulated pathways (bottom), compared to total background detection for ramipril (B) and quinapril (C). Classification stringency set to ‘medium’, similarity threshold=0.5. Bars represent enrichment score and points (•) are the number of proteins detected in that pathway. *P*-values are presented.

Analysis of structural similarities suggests that a number of drugs may have therapeutic actions similar to ACE-Is. Therefore, we sought to elucidate the protein pathways that are dysregulated by ACE-I treatment in our model. Peptide samples from dissected head tissue from ACE-I-treated ICH+ larvae were analysed using tandem mass spectrometry (MS/MS). Normalised abundances for each sample (Fig. S1) were used to determine the log2 fold change in expression from DMSO ICH+ controls. Proteins identified from MS/MS analysis as being upregulated (log2>0.8) (ramipril, 191; quinapril, 304) or downregulated (log2<−0.5) (ramipril, 602; quinapril, 376) are presented in Fig. 3A. Cut-off parameters were adjusted to allow for an overall greater number of downregulated proteins. Protein lists were analysed for significant pathway enrichment to identify potential mechanisms for neuroprotection. The list of total detected proteins was used as a background control, and pathway enrichment for each drug condition is presented in Fig. 3B and C. The most significant pathways suggest that ACE-I treatment influences protein synthesis and transport, functionality of the BBB, and changes to cellular metabolism and mitochondrial function. Most significantly upregulated pathways with both drug treatments include the ribosome pathway, suggesting that protein synthesis is a key function of neuroprotection in this system. The full proteomics data set is available on Figshare (doi:10.6084/m9.figshare.14096543).

### Analysis of the INTERACT2 dataset shows that ACE-I treatment only after ICH is associated with good functional outcome

**Fig. 4.**
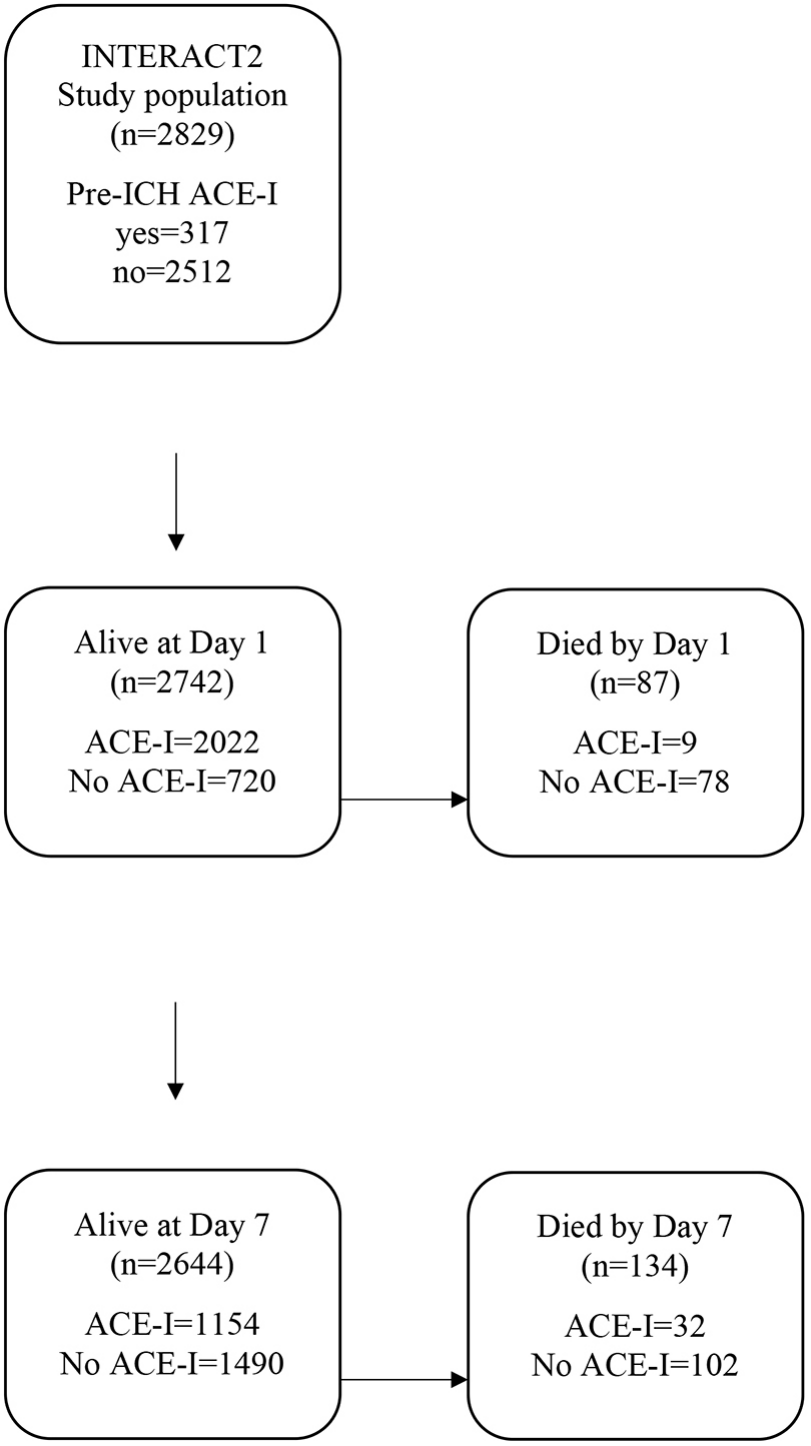
**Flowchart showing exposure to ACE-I at onset, day 1 and day 7 in patients participating in the INTERACT2 trial.** Exposure to ACE-I is shown separately for those alive and dead at day 1 and day 7.

**Fig. 5.**
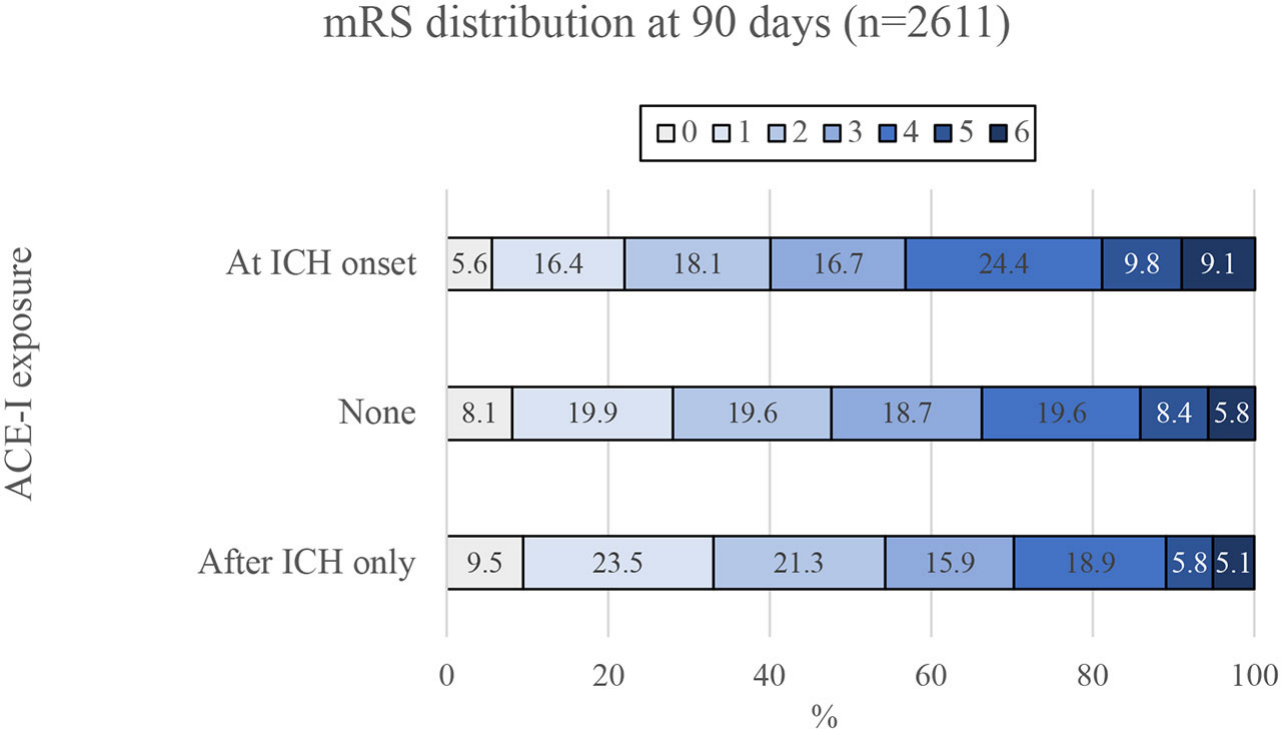
**ACE-I given after ICH is associated with improved modified Rankin scale (mRS) scores at 90 days.** Exposure to an ACE-I after ICH only, and within 7 days of ICH (bottom) [versus no ACE-I exposure (middle)], was associated with a favourable shift in functional outcomes in a multifactorial ordinal shift analysis (*P*=0.009). Exposure to an ACE-I at ICH onset (top, versus no ACE-I exposure) was not independently associated with a shift in the mRS score at 90 days (*P=*0.169).

Following positive results in the zebrafish model with ACE-I treatment, we analysed existing clinical trial data to identify a potential translational impact. Given that ACE-Is are already commonly prescribed to ICH patients to control blood pressure, we sought to test whether exposure to an ACE-I in the first 7 days after ICH is associated with early neurological recovery by day 7 and/or functional outcome at day 90 in patients taking part in the INTERACT2 trial (Anderson et al., 2013). We also tested for an association between exposure to an ACE-I by day 1 and oedema and/or haematoma expansion by 24 h (Fig. 4). For analysis of neurological recovery and functional outcome, we excluded 228 patients who had died by day 7, leaving 2611 patients for our main analysis. Of these, 1030 participants had been exposed to an ACE-I only after the ICH and within 7 days of onset, 1294 received no ACE-I, and 287 were taking an ACE-I at the time of ICH onset (Fig. 4, Table 2). After adjusting for potential confounders (Table 3), we found that exposure to an ACE-I after the ICH only (versus no exposure) was significantly associated with a favourable shift in scores on the modified Rankin scale (mRS) at 90 days in a multifactorial ordinal regression analysis [adjusted odds ratio (OR) for an unfavourable shift in mRS, 0.80; 95% confidence interval (CI), 0.68-0.95; *P*=0.009] shown in Fig. 5. To ensure that exclusion of patients that died by day 7 did not alter our main result, we conducted a sensitivity analysis, this time including these patients. This led to a similar result (OR, 0.77; 95% CI, 0.65-0.90; *P*=0.001). There was no significant association between ACE-I exposure at ICH onset (versus no exposure) and 90 day mRS. No significant associations were found for ACE-I exposure and other outcomes, including change in scores on the National Institutes of Health Stroke Scale (NIHSS) from baseline to day 7, change in oedema extension distance (Parry-Jones et al., 2015) from baseline to 24 h, and haematoma expansion (defined as >33% and/or >6 ml) from baseline to 24 h.

**Table 2.**
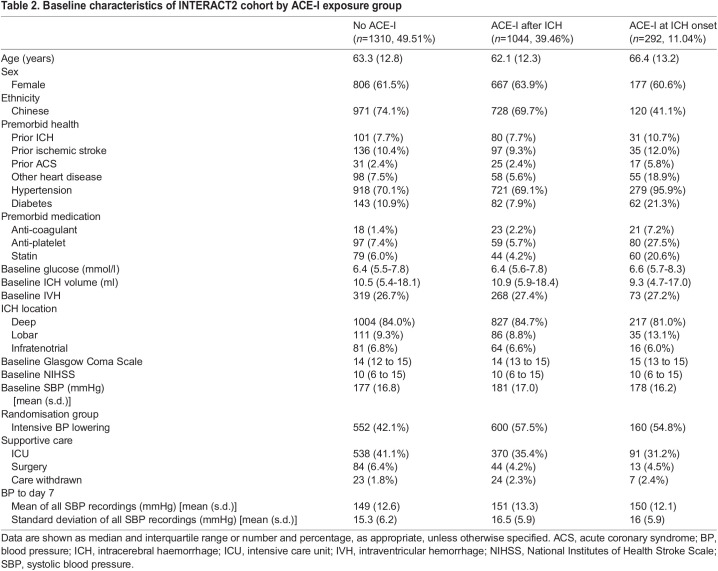
Baseline characteristics of INTERACT2 cohort by ACE-I exposure group

**Table 3.**
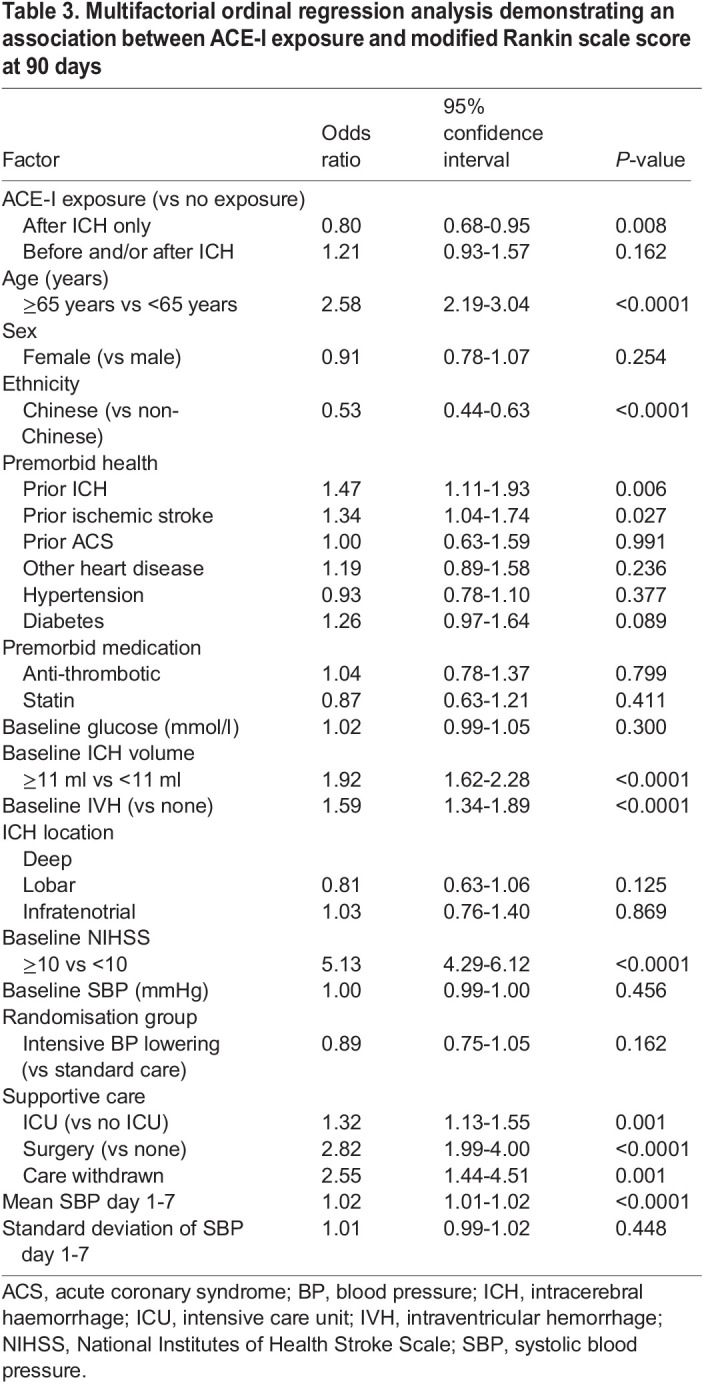
Multifactorial ordinal regression analysis demonstrating an association between ACE-I exposure and modified Rankin scale score at 90 days

## DISCUSSION

Drug repurposing for ICH patients offers the most immediate translatable treatment potential (Crilly et al., 2021). Herein, we report our investigation of a large-scale drug screen that, to the best of our knowledge, has never before been carried out in a pre-clinical animal model for ICH. This screen identified both potentially repurposable drugs in addition to compounds not investigated in humans before with limited experimental data. As such, our dataset of 150 compounds provides a resource of candidate repurposable/organic drugs ready for further interrogation by the wider ICH research community.

ACE-Is are currently recommended for the treatment of hypertensive ICH patients, primarily for secondary prevention and regulation of co-morbid hypertension (Dastur and Yu, 2017). Based on our zebrafish drug screen, we propose that ACE-Is also have specific neuroprotective properties and hold the potential to reduce disabilities in patients when administered after ICH has occurred. Previous pre-clinical studies have also shown the benefit of ACE-Is on stroke outcomes mediated by a reduction in blood pressure (Vacher et al., 1993). Clinical trials show that ramipril reduced stroke risk in high-risk cardiovascular patients (Sleight, 2000). An ACE-I trial in stroke patients suggested that ACE-Is or angiotensin II receptor blockers in ICH patients result in less peri-haematomal oedema at 3 days, improve 3 month mortality and reduce rate of post-stroke pneumonia (Zhang et al., 2021). The authors of this trial hypothesised that anti-inflammation is the mechanism of protection rather than direct blood pressure lowering; however, our proteomics data suggest that there is no direct modulation of inflammatory pathways with ACE-I treatment. Our analysis of the INTERACT2 trial data suggests that ACE-Is may offer protection when administered within 7 days after an ICH, although our secondary analysis of the dataset is observational and the patients were not randomised. Although we adjusted for multiple key prognostic factors, we cannot discount the possibility that unmeasured confounding factors may account for our results. However, these data do suggest that if ACE-Is could be administered in an alternative formulation, to avoid the limitation of patient swallowing, this would increase accessibility at earlier phases of pathology. In support of our findings, Eichel et al. suggested that patients taking ACE-Is prior to ICH exhibit no neuroprotection or prevention of mortality as a result of treatment (Eichel et al., 2010); however, the small number of patients included in this analysis may not conclude a definitive lack of association. This analysis of clinical research raises a translational caveat, as prospective ICH patients are more likely to be hypertensive and receiving blood pressure-lowering treatment such as ACE-Is prior to an ICH event, and evidence suggests that these patients would not benefit from any potential neuroprotection. Further clinical studies, and pre-clinical studies in models of co-morbidity, are required to determine the importance of formulation, timing and dose or ACE-I therapy.

Interestingly, from the MACCS structural key similarities, the three closest compounds to ramipril and quinapril hydrochloride ACE-Is are lappaconitine, lincomycin hydrochloride and cyclosporine (Fig. 1B). Lappaconitine, a plant-derived diterpenoid alkaloid, acts as a natural analgesic and a sodium channel blocker on the heart muscle, reducing heart rate and blood pressure. Hypertension is the most significant risk factor for ICH, and elevated blood pressure (>220 mmHg) is associated with poorer neurological outcomes (Qureshi et al., 2020). Lowering of blood pressure following ICH (<140 mmHg) has been trialled clinically in order to prevent haematoma expansion and limit brain damage caused by cerebral oedema (Anderson et al., 2013; Qureshi et al., 2016). These studies (INTERACT2 and ATACH2) conflicted in their conclusion of the benefit from intensive blood pressure lowering; thus, it has become clear that the type of medication used and timing of delivery are also important factors to consider (Rabinstein, 2018). Lincomycin, an antibiotic in use for penicillin allergies, has not been linked to the cardiovascular system, with the exception of arrhythmias being a notable rare side effect. Cyclosporine is an immunosuppressive, trialled in ischemic stroke patients with no additional benefits to thrombolysis (Nighoghossian et al., 2015). There is also evidence to show that cyclosporine can stabilise mitochondria in cerebral ischemia models (Forsse et al., 2019), which may therefore suggest a neuroprotective mechanism. The compounds that are also closely related in structure to the other four drugs indicated to be effective in cardiovascular disease may also provide a novel route for pre-clinical investigation.

In this study, we have only pursued ACE-I therapy due to current clinical interest; however, evidence suggests that the other four compounds identified from our screen may also offer viable repurposable treatment options. Digoxin is a cardiac glycoside prescribed for maintenance of atrial fibrillation. It inhibits membrane sodium–potassium pumps to increase cytoplasmic calcium and promote cardiac contractility and vasoconstriction, although it does not affect blood pressure, but has been associated with a higher risk of ischemic stroke (Lai et al., 2018). No causative or protective relationship has yet been identified between treatment for cardiac arrhythmia and ICH (Nagarakanti et al., 2016). Disopyramide is also prescribed for cardiac arrhythmia and blocks sodium channels, thus decreasing contractility in the myocardium. By regulating the circulation, treatment decreases the risk of blood clots and indirectly reduces the risk of stroke; however, there has been no direct relationship made between anti-arrhythmia drugs and stroke outcome. Clonidine, a centrally acting anti-hypertensive drug that blocks adrenergic activation, may reduce inflammation after ICH and peri-haematomal oedema (Sansing et al., 2011). In a small ischemic stroke trial, 0.1 mg clonidine administered every 8 h for 3 days resulted in a drop in blood pressure but no impact on stroke outcomes (Lisk et al., 1993). INTERACT researchers reported clonidine as a blood pressure-reducing agent; however, the number of patients taking clonidine was <4% of those enrolled and so a relationship is inconclusive (Anderson et al., 2008). Acamprosate has proven safe and effective for alcohol addiction in clinical trials due to a neuromodulatory effect and neuroprotection through indirect antagonism of NMDA receptors (Mason and Heyser, 2010). In a pre-clinical model of cerebral ischemia, acamprosate proved to be directly neuroprotective when administered before (Engelhard et al., 2000) and repeatedly after stroke (Doeppner et al., 2015), with reduced infarct size, cerebral oedema, increased neuronal density and ameliorated tissue-plasminogen activator-induced toxicity. This screen has revealed that these drugs, and the other 150 identified compounds, may have promise in medical treatment of ICH. Further investigation could reveal a treatment strategy for patients through repurposing these neuroprotective drugs.

Proteomic analysis carried out in this study highlights some potential mechanisms by which ACE-Is are neuroprotective in the zebrafish ICH model. The top ten significant pathways highlighted by enrichment analysis are shown in Fig. 3. Ribosomal protein pathways, protein processing and the ErbB signalling pathway of proliferation all suggest that neuroprotection may occur through upregulation of neuronal protein synthesis and proliferation. Some pathways shown to be upregulated, such as ECM–receptor interactions, focal adhesions, protein processing and tight junction-specific proteins, could imply a change in the structure of the endothelial tight junctions in the BBB. Improved functionality of the BBB after ICH could contribute to less inflammation and extravasation of peripheral immune cells and a decrease in cerebral oedema shown to benefit functional outcome (Keep et al., 2008). Mitochondrial proteins were the most downregulated with both ACE-I treatments. Mitochondria have been considered potential therapeutic targets for the treatment of many different brain diseases through the inhibition of oxidative stress (Chaturvedi and Beal, 2008; Guo et al., 2013). Similarly, evidence suggests that modulating mitochondrial action could be therapeutic in cardiovascular diseases (De Cavanagh et al., 2007; Henriksen and Jacob, 2003; Zoll et al., 2006). Most recently, ACE-Is as renin–angiotensin system inhibitors were identified to be directly neuroprotective in a zebrafish model for Parkinson's disease (Kim et al., 2021). As such, we hypothesise that downregulation of mitochondrial proteins by ACE-Is could offer neuroprotection through prevention of the dysregulation associated with reactive oxygen species release and mitochondrial DNA mutations. Further detailed interrogation of the potential protective mechanisms of ACE-Is in ICH will form the basis of future studies.

Prevention of apoptosis in brain cells is not the only pathological outcome from ICH, and we recognise the limitations to our drug screen methodology. Our positive hits may only be effective inhibitors of apoptosis, and there are many different injury mechanisms occurring within the haematoma and the surrounding tissue (Zille et al., 2017). Additionally, measures of the dying tissue are not directly made in patients, and therefore we can only draw an associated link with functionality. However, we propose that these cells in the peri-haematomal region have the potential to be rescued after injury and are of most interest therapeutically. Another limitation of this model is that intracranial pressure, increases in which are associated with worse prognosis after ICH, cannot be measured easily and would be influenced by blood pressure-lowering drugs (Yang et al., 2015).

This study further validates zebrafish larval models as important drug discovery tools. Fundamentally, a screen of this type in a spontaneous model of ICH has never been possible before, and the zebrafish model offers unique advantages for the translational ICH research community. The positive drug hits we present here may offer a new direction of investigation for the medical treatment of ICH. We have provided the full results of the drug screen in the hope that this will support further pre-clinical study and drive translation for ICH therapies.

## MATERIALS AND METHODS

### Study design

The 2000-drug Spectrum Collection library was screened in 96-well plates using the zebrafish larval model of spontaneous ICH and live microscopy. Drugs that had a positive outcome on brain cell death were investigated twice. From 150 positive drug hits, six were predicted to work in cardiovascular disease using PREDICT and MBiRW algorithm datasets (Table 1) (Gottlieb et al., 2011; Luo et al., 2016). Two drugs out of the final six were the ACE-Is ramipril and quinapril, and these drugs were interrogated in larger sample sizes to verify brain cell protection and to determine effects on locomotor function. Mass spectrometry and proteomics were used to identify pathways associated with neuroprotection following ACE-I treatment. Analysis of the INTERACT2 trial data was carried out to determine a translatable effect.

### Zebrafish husbandry

Zebrafish (*Danio rerio*) were raised and maintained at The University of Manchester Biological Services Unit under standard conditions as previously described (Westerfield, 2000). Adult zebrafish husbandry was approved by The University of Manchester Animal Welfare and Ethical Review Board. All experiments were performed in accordance with the UK Home Office regulations (PPL: P132EB6D7) and reported according to the ARRIVE guidelines (https://arriveguidelines.org/arrive-guidelines). Transgenic zebrafish used for this study are *ubiq*:secAnnexinV-mVenus, a fluorescent reporter for cell death [re-derived in house (Morsch et al., 2015)] on a mutant *bbh* (*bbh*^m292^) or nacre (*mitfa*^w2/w2^) background (herein, *bbh* annexin). Adults were selectively bred using isolated boxes, and fertilised embryos were collected and incubated at 28°C in standard E3 medium and staged according to standard guidelines. Zebrafish larvae were terminated prior to protected status with an overdose of 4% MS222 anaesthesia and freezing at −20°C.

### Spectrum Collection library screening

The Spectrum Collection drug library (MicroSource Drug Systems Inc.) consisting of 25×96-well plates with 2.5 µl of 2.5 mM drugs in DMSO was purchased from The University of Sheffield. Fertilised larvae from homozygous *bbh* annexin adults and annexin wild-type controls were collected and dechorionated at 24 hpf. Larvae were confirmed for haemorrhage (ICH+/ICH−) at 48 hpf and plated (*n*=3) into 96-well plates containing 25 µM drug in 1% DMSO diluted in E3 medium without Methylene Blue. Control ICH− annexin larvae were plated in 1% DMSO only. Plates were incubated for 24 h, and, at 72 hpf, larvae were anaesthetised briefly and screened for overall health, cerebral oedema and characteristic clusters of annexin-positive cells in the brain using a Leica M165FC fluorescent stereomicroscope. Representative images were acquired using the DFC700T camera and LAS-X software (version 3.3.3.16958). Positive drug hits were repeated at 25 µM with *n*=5 larvae in 150 µl volume in E3 medium to confirm neuroprotection and are listed in the full dataset doi:10.6084/m9.figshare.14096465.

### Visualising drug screen results

Successful drugs (150 compounds) were analysed using the cSPADE web tool (Ravikumar et al., 2017) to visualise structural similarities. Simplified molecular input line-entry system (SMILES) data were acquired for each compound with the exception of bephenium hydroxynapthoate, for which no data are available. This list was uploaded to the cSPADE website (https://cspade.fimm.fi/; Ravikumar et al., 2017), and, using MACCS structural keys, similarities between compounds were visualised using a radial tree.

### Predicting drug/disease interactions

DrugBank numbers were acquired for the drugs identified as successful. Using the Online Mendelian Inheritance in Man (OMIM) search terms ‘haemorrhage’, ‘cerebral hypertension’ and ‘cerebrovascular’, numbers for intracerebral haemorrhage related diseases were identified (Table S1). The PREDICT and MBiRW databases of predicted drug/disease interactions were searched for drugs identified in the screen and any OMIM numbers identified in Table S1, implying a predicted interaction with these drugs and cerebrovascular disease.

### Analysis of disease pathology of ICH following ACE-I treatment

Heterozygous *bbh* annexin fish were in-crossed for a population of experimental larvae. ICH− and ICH+ populations were isolated at 48 hpf and plated in treatment plates (*n*=30) containing 25 µM of each ACE-I in 1% DMSO. Images of cell death were collected using light-sheet microscopy as previously described at 72 hpf, and blinded prior to analysis as previously stated (Crilly et al., 2018). Locomotor function was analysed at 120 hpf as previously described (Crilly et al., 2018). Larvae were terminated prior to protected status at 126 hpf using a lethal overdose of anaesthesia (4% MS222).

### Sample preparation for proteomics

At 72 hpf, heads were dissected from ICH+ larvae treated for 24 h with ramipril, quinapril or a DMSO control (*n*=5). Samples were lysed in S-Trap buffer and sonicated using a Covaris LE220+ system at 500 W for 8 min. Proteins were reduced, alkylated and digested using the standard S-Trap column protocol and trypsin digestion enzyme at 2 µg/µl. Peptides were eluted into a solution of 30% aqueous acetonitrile with 0.1% formic acid. Peptides were desalted using POROS R3 beads and eluted into a final solution of 30% aqueous acetonitrile with 0.1% formic acid. Samples were completely dried in a Heto speed vacuum centrifuge.

### Mass spectrometry

Digested samples were analysed by liquid chromatography (LC)-MS/MS using an UltiMate^®^ 3000 Rapid Separation LC (RSLC, Dionex Corporation, Sunnyvale, CA, USA) coupled to a QE HF (Thermo Fisher Scientific, Waltham, MA, USA) mass spectrometer. Mobile phase A was 0.1% formic acid in water, mobile phase B was 0.1% formic acid in acetonitrile, and the column used was a 75 mm×250 μm i.d. 1.7 µM CSH C18 analytical column (Waters).

A 1 μl aliquot of the sample was transferred to a 5 μl loop and loaded on to the column at a flow of 300 nl/min for 5 min at 5% B. The loop was then taken out of line, and the flow was reduced from 300 nl/min to 200 nl/min in 0.5 min. Peptides were separated using a gradient that went from 5% to 18% B in 63.5 min, then from 18% to 27% B in 8 min and finally from 27% B to 60% B in 1 min. The column was washed at 60% B for 3 min before re-equilibration to 5% B in 1 min. At 85 min the flow was increased to 300 nl/min until the end of the run at 90 min. Mass spectrometry data were acquired in a data-directed manner for 90 min in positive mode. Peptides were selected for fragmentation automatically by data-dependent analysis on a basis of the top 12 peptides with m/z between 300 and 1750 Th and a charge state of 2, 3 or 4 with a dynamic exclusion set at 15 s. The MS resolution was set at 120,000 with an automatic gain control (AGC) target of 3×10^−6^ and a maximum fill time set at 20 ms. The MS2 resolution was set to 30,000, with an AGC target of 2×10^−5^, a maximum fill time of 45 ms, isolation window of 1.3Th and a collision energy of 28.

### Analysis of mass spectrometry data

The raw data files from the instrument were processed using Proteome Discoverer (PD) (version2.3). The data were processed using the consensus workflow provided with PD in the file CWF_Comprehensive_Enhanced_Annotation_LFQ_and_Precursor_Quan and the processing workflow provided with PD in the file PWF_QE_Precursor_Quan_and_LFQ_SequestHT_Percolator. The processing workflow was set to search the SwissProt *Danio rerio* protein database (v2021-02-04) and TrEMBL *Danio rerio* protein database (v2021-02-04) (https://www.uniprot.org/) using the Sequest protein identification search engine algorithm provided with PD. The protein identification algorithm was provided with trypsin as the cleavage enzyme cleaving at lysines and arginines except where the presence of a C-terminal proline obstructs cleavage. Two missed cleavage events were permitted. The algorithm searched for a fixed modification of carbamidomethyl (+57.021 Da) to cysteines and a dynamic modification of oxidation (+15.995 Da) to methionines. A precursor tolerance of 10 ppm and a fragmentation tolerance of 0.02 Da was used. A false discovery rate (FDR) was calculated for both the protein level and the peptide level by PD. Proteins were labelled with high confidence where the FDR was less than 0.01, medium confidence where the FDR was between 0.01 and 0.05, and low confidence where the FDR was greater than 0.05. Samples were grouped according to the experimental conditions of DMSO control, and ramipril and quinapril treated. Ratios were generated for all conditions to the DMSO condition. Proteins that were significantly (*P* adjusted≤0.05) up/downregulated in both conditions were assessed for pathway enrichment using FishEnrichr (https://maayanlab.cloud/FishEnrichr/) and the Kyoto Encyclopedia of Genes and Genomes (KEGG) database (2019). Raw data can be accessed on Figshare (ICH_ACEI_MS doi:10.6084/m9.figshare.14096543).

### Analysis of INTERACT2 trial data

The INTERACT2 trial was a phase III randomised controlled trial of intensive blood pressure lowering in acute ICH (Anderson et al., 2013). The INTERACT2 trial grouped all medical blood pressure-lowering strategies together; however, we undertook secondary analysis of the INTERACT2 dataset to determine the effects of ACE-I only. Patients who died by day 7 were excluded, and all remaining patients were included in the analysis of mRS at 90 days and NIHSS at day 7. Our rationale for excluding patients who died before day 7 was that there will have been less time for them to be exposed to an ACE-I; thus, we expect them to be over-represented in the group not taking an ACE-I after ICH, introducing potential bias. For analyses of oedema and haematoma expansion by 24 h, we excluded patients who had died by day 1. Exposure to an ACE-I was captured in INTERACT2 at baseline (that is, medications at time of admission), day 1 and day 7. As many ICH patients are not established on enteral medication on day 1 due to impaired swallow, we chose day 7 to look for exposure to an ACE-I after ICH. Patients were thus categorised into three groups with respect to ACE-I exposure: (1) those who had no exposure to an ACE-I, (2) those exposed to ACE-I after ICH only (commenced an ACE-I by day 7 after onset but not before the ICH), and (3) those taking an ACE-I at ICH onset. This allowed testing for associations between exposure to an ACE-I at ICH onset (versus no ACE-I exposure) and exposure to an ACE-I only after onset (versus no ACE-I exposure). Status with regard to ACE-I exposure was included in an appropriate multifactorial ordinal or linear regression model, conducted in SAS, to test for independent associations with outcomes, including mRS at day 90, change in the NIHSS score from baseline to day 7, haematoma expansion (>33% and/or <6 ml) and change in oedema extension distance from baseline to 24 h computed tomography brain scans. We adjusted for demographics, co-morbidities, pre-ICH medications, glucose on arrival, ICH severity, trial randomisation group, supportive care, and the mean and s.d. of systolic blood pressure from 15 min post-randomisation to day 7. All baseline characteristics are in Table 2. Age, ICH volume and NIHSS were entered into the models as binary variables, using medians as cut-off values.

### Statistical analysis

Experimental sample sizes were determined using power calculations from preliminary data using α=0.05 and β=0.80. All statistical analysis was performed using GraphPad Prism 7.0 for parametric datasets and R (https://www.r-project.org/) for non-parametric data (Fig. 2) using generalised linear mixed modelling to evaluate the effects of independent factors on the continuous dependent variable with subsequent significant values plotted (Crilly et al., 2018) (Dataset 1).

## Supplementary Material

Supplementary information
