# Supplementary material for: Zebrafish drug screening identifies candidate therapies for neuroprotection after spontaneous intracerebral haemorrhage
Source: Dis Model Mech. 2022 Mar 29;15(3):dmm049227. doi: 10.1242/dmm.049227 (PMC8990924; doi:10.1242/dmm.049227)
Supplement: Supplementary information [file dmm-15-049227-s1.pdf]

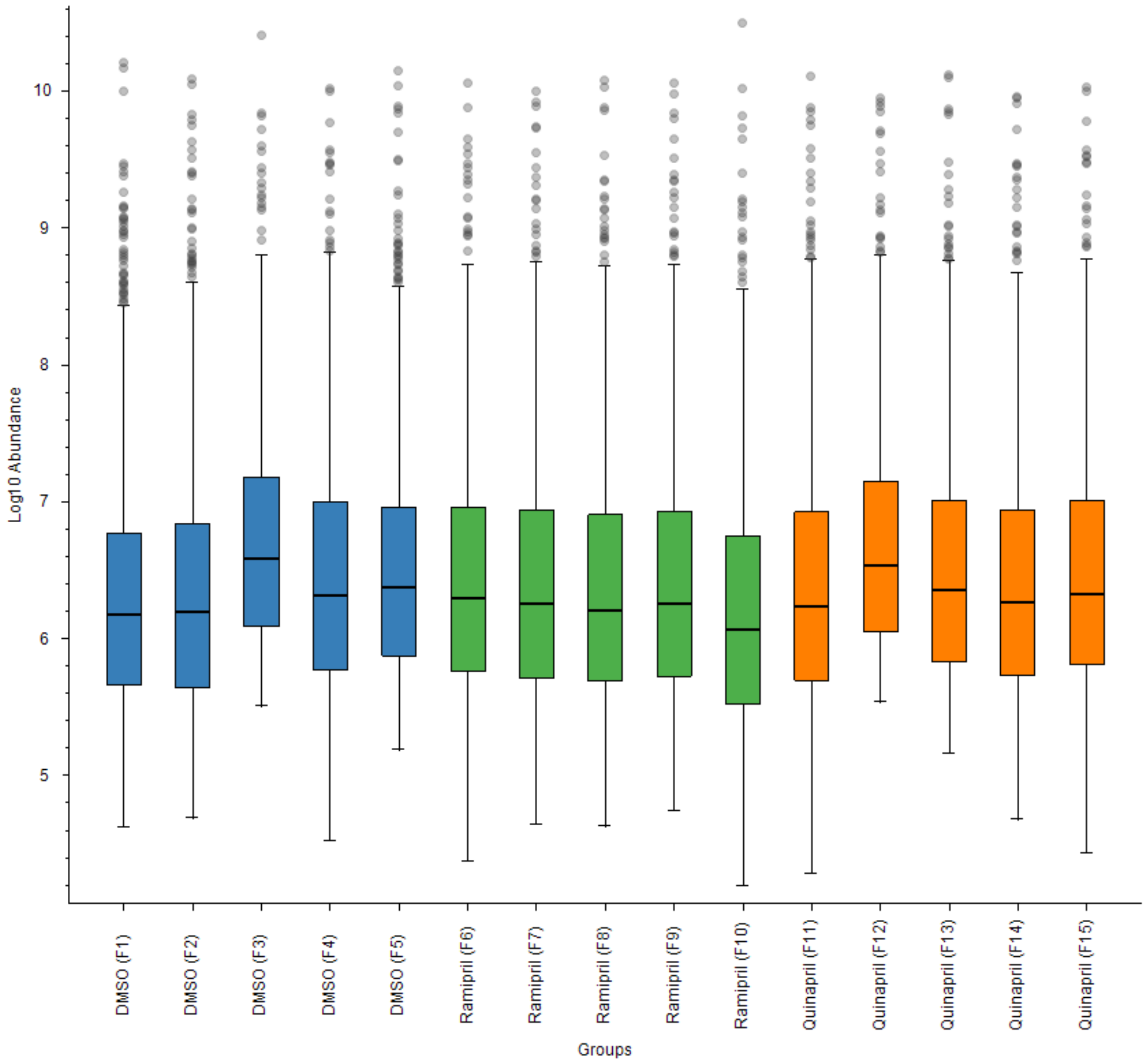

Fig. S1. Normalized abundances of mass spectrometry samples. Box and whisker plots represent the median and inter-quartile range.

Disease Models & Mechanisms • Supplementary information

Table S1. OMIM numbers for cerebrovascular disease search terms used in PREDICT and MBiRW

**Disease**	**OMIM**
Aicardi-Goutieres Syndrome (AGS)	225750
Aneurysm	210050<br>611773<br>614816<br>618734
Basal ganglia calcification	213600<br>114100
Brain haemorrhage	108010<br>614519<br>613730
Brain small vessel disease	175780<br>618360<br>614483
CADASIL	125310<br>616779
CARASIL	600142
Cerebral amyloid angiopathy (CAA)	605714<br>137440<br>105150<br>117300<br>176500
Cerebral arteriovenous anomaly	152900<br>108010
Cerebral cavernous malformations (CCM)	116860<br>603285
Col4a2/Col4a1	120090<br>120130<br>618564<br>611773
Hereditary haemorrhagic telangiectasia (HHT)	600376<br>187300<br>610655
Homocystinuria	236200<br>603174
Ischemic stroke	601367
Moyamoya	300845<br>252350<br>614042<br>607151<br>615750
Notch3	600276

Dataset 1. Script for non-parametric statistical testing for motility data and plots of significant values

R Script for “motility ACEI.csv”

‘Factor 1’ is haemorrhage

‘Factor 2’ is treatment

```r
#Load dataset
Data<-read.csv("P:/Documents/motility ACEI.csv")
str(Data)

library(multcompView)
library(lsmeans)
library(emmeans)
library(lme4)
library(lmerTest)
library(ggplot2)


lme1<-lmer(Data~1 + (1|Clutch), Data, na.action=na.omit)
lme2<-update(lme1, .~. +Factor.1)
lme3<-update(lme2, .~. +Factor.2)
lme4<-update(lme3, .~. +Factor.2*Factor.1)

aov=anova(lme1,lme2,lme3,lme4)

par(mfrow=c(2,2))
plot(aov)

plot(lme4,col=Data$Factor.1,pch=as.numeric(Data$Clutch)+14, ces=0.6)
qqnorm(resid(lme4))
augDat <- data.frame(data,resid=residuals(lme4,type="pearson"),
                     fitted=fitted(lme4))
ggplot(augDat,aes(x=Factor.1,y=resid,col=Clutch))+geom_point()+geom_boxplot(aes(group=Factor.1),alpha = 0.1)+coord_flip()
```

```
Data: Data
Models:
lme1: Data ~ 1 + (1 | Clutch)
lme2: Data ~ (1 | Clutch) + Factor.1
lme3: Data ~ (1 | Clutch) + Factor.1 + Factor.2
lme4: Data ~ (1 | Clutch) + Factor.1 + Factor.2 + Factor.1:Factor.2
     npar    AIC    BIC  logLik deviance   Chisq Df Pr(>Chisq)
lme1    3 2284.3 2294.1 -1139.2   2278.3
lme2    4 2274.4 2287.3 -1133.2   2266.4 11.9594  1  0.0005437 ***
lme3    6 2277.4 2296.8 -1132.7   2265.4  1.0104  2  0.6033701
lme4    8 2279.5 2305.4 -1131.7   2263.5  1.9042  2  0.3859288
---
Signif. codes:  0 ‘***’ 0.001 ‘**’ 0.01 ‘*’ 0.05 ‘.’ 0.1 ‘ ’ 1
>
```

Effect of random variable (clutch)

Disease Models & Mechanisms • Supplementary information

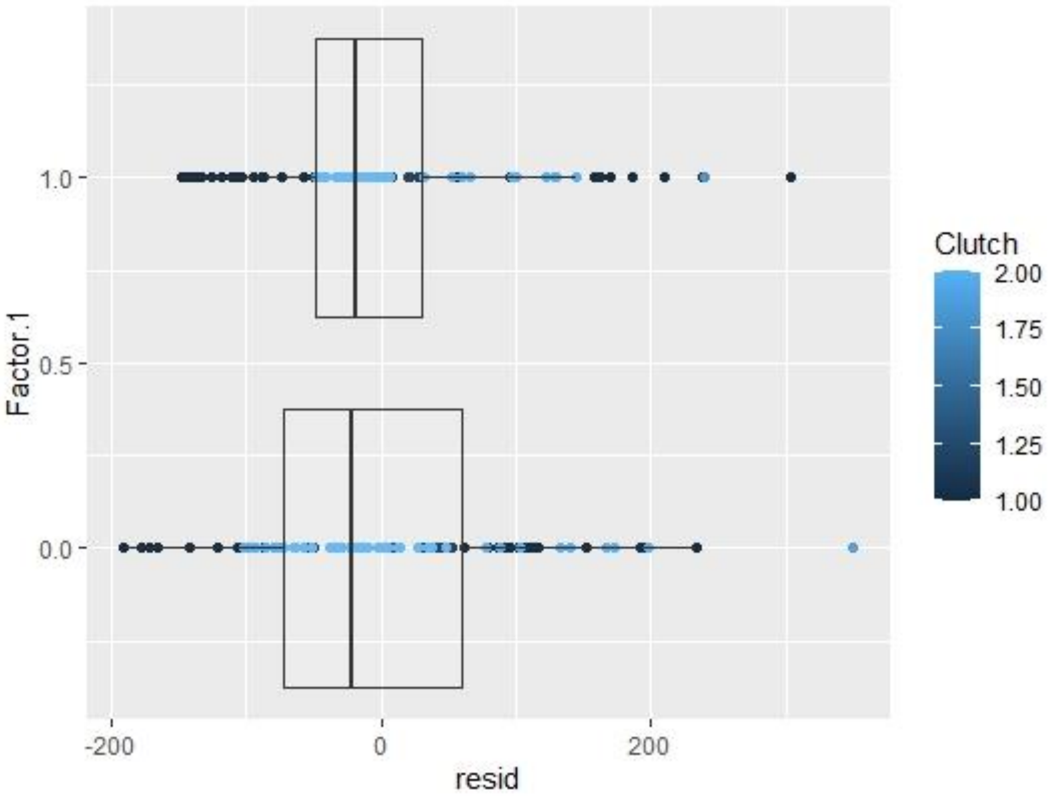

Effect of drug

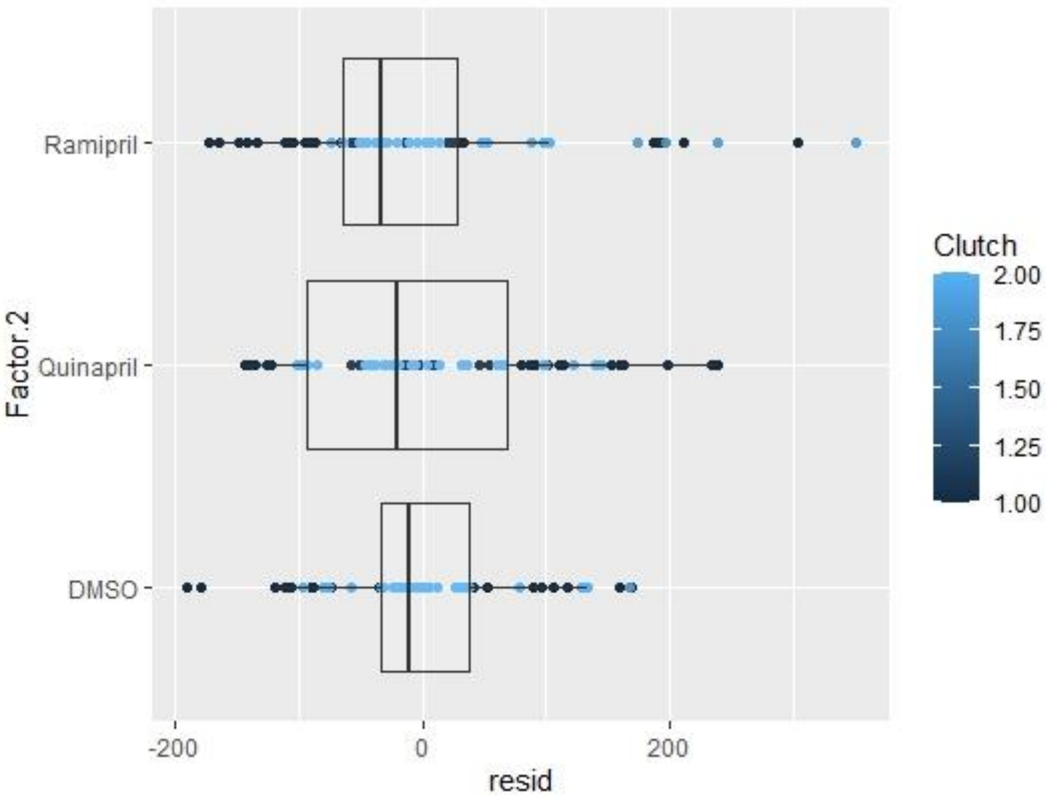

Pearson

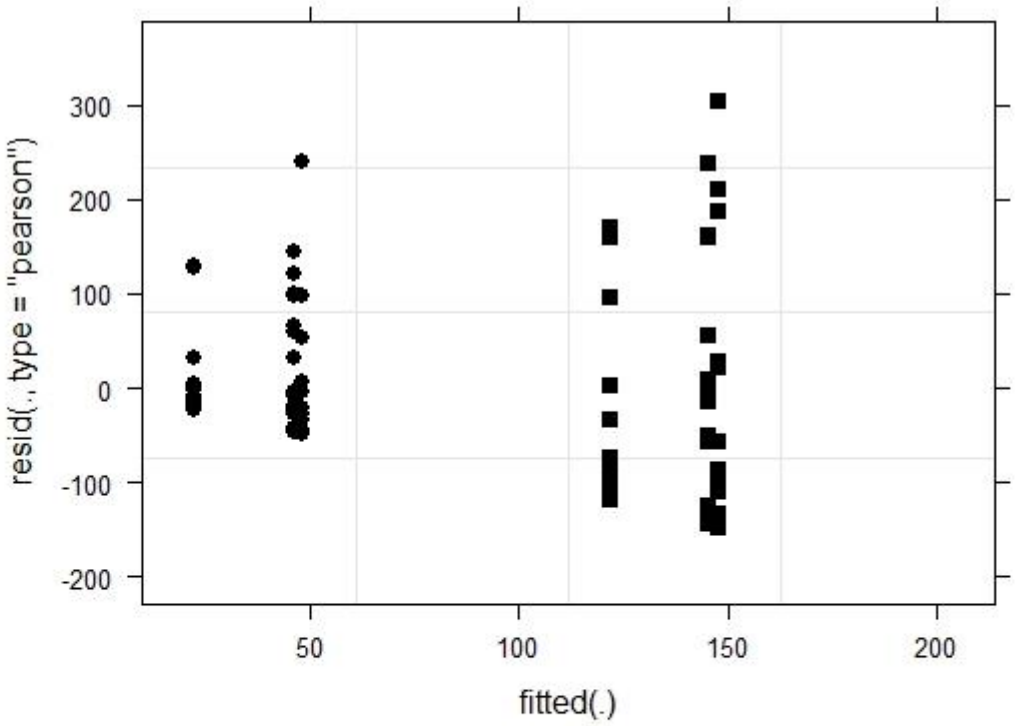

Q-Q

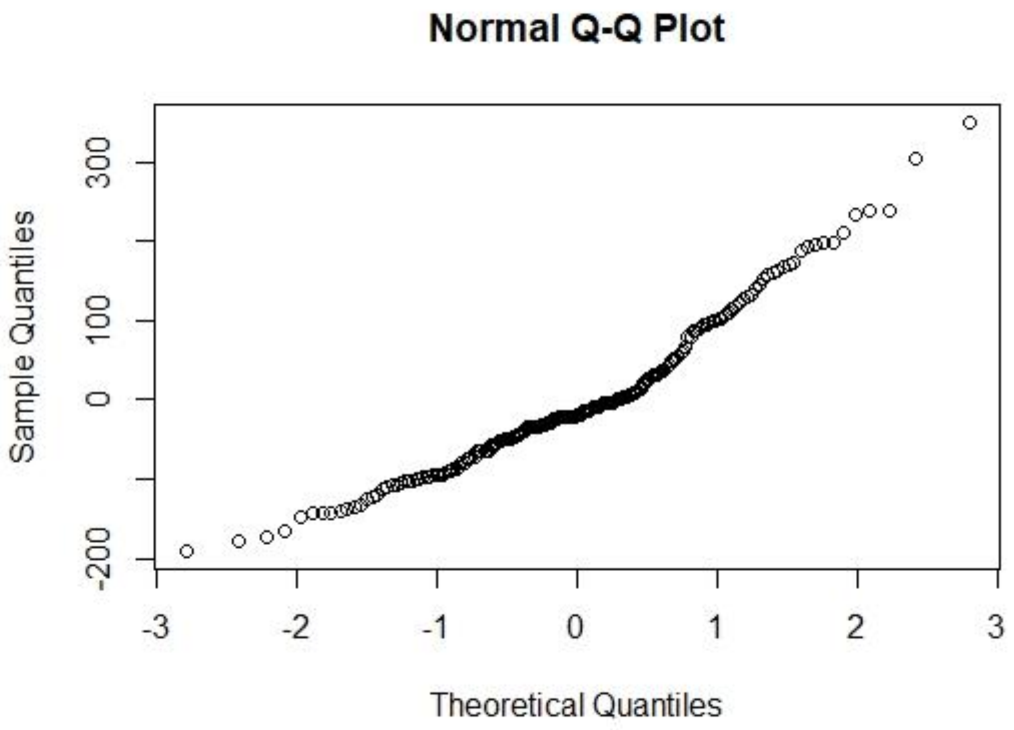

Disease Models & Mechanisms • Supplementary information
